# Advancing the frontiers of geographic accessibility to healthcare services

**DOI:** 10.1038/s43856-023-00391-w

**Published:** 2023-11-03

**Authors:** Peter M. Macharia, Aduragbemi Banke-Thomas, Lenka Beňová

**Affiliations:** 1 Department of Public Health, Institute of Tropical Medicine, Antwerp, Belgium; 2 Population & Health Impact Surveillance Group, Kenya Medical Research Institute-Wellcome Trust Research programme, Nairobi, Kenya; 3 Faculty of Epidemiology and Population Health, London School of Hygiene and Tropical Medicine, London, UK; 4 Maternal and Reproductive Health Research Collective, Lagos, Nigeria; 5 School of Human Sciences, University of Greenwich, London, UK

**Keywords:** Epidemiology, Epidemiology

## Abstract

Macharia et al. discuss a *Communications Medicine* article on global healthcare accessibility and the impact of the COVID-19 pandemic. They outline strengths in the comprehensive approach taken to studying revealed versus potential spatial accessibility, plus some limitations and wider context with which the results can be interpreted.

## Teaser

Assessing geographic accessibility to healthcare is essential to identify communities that have been left behind. Smartphone mobility data now enables the study of healthcare accessibility over a global scale, providing estimates of actual travel times to access care.

During the International Conference on Primary Health Care in Kazakhstan in 1978, the Alma-Ata Declaration endorsed by World Health Organization (WHO) member states identified primary health care as key to achieving health for all^1^. Countries renewed their commitment to universal health coverage (UHC) during the Global Conference on Primary Health Care in 2018 in the Declaration of Astana^2^. The Sustainable Development Goal target 3.8 aims to ensure comprehensive and quality health services to individuals without financial burden and underpins the global commitment to achieve UHC^3^. Evidence shows that better access to healthcare is associated with improved health outcomes, including morbidity and mortality^4,5^. Therefore, it is essential to periodically evaluate a population’s healthcare access to identify those left behind to ensure their prioritization during decision-making and future resource allocation.

Broadly, healthcare access is the outcome of interactions between provision of healthcare, needs of the population, and public policy and planning efforts^6–9^. According to Penchansky and Thomas, healthcare access is multi-dimensional, encompassing availability, accessibility, affordability, acceptability and accommodation (arrangement and organization of health services to meet population demand)^6^. Access can be spatial (geographic) or aspatial (social). Aspatial access is conditioned by non-geographic barriers or facilitators such as social status, income, ethnicity, and sex^8,10^. Spatial access (proximity between people and healthcare services) can either be potential (where people have the option of seeking care) or revealed (where people actually sought care). The choice of either potential or revealed as a measure of spatial accessibility is a function of the research question, technical expertise and ease of implementing a certain method, area (rural or urban), size of the geographical area and its features (topography), context (socio-economic status, ethnic composition, and cultural diversity) and data availability. Advances in geospatial technologies such as global navigation satellite systems, geographical information systems and earth observation have led to a proliferation of studies on potential access^4,11–15^.

In an article now published in *Communications Medicine*, Gligorić et al. present an exciting study of revealed and potential spatial access in addition to answering four interconnected questions on healthcare access in over 120 countries globally^16^. To do so, they assembled a database of health facilities comprising hospitals and medical centres capable of urgent and emergency care. First, they estimated potential spatial accessibility to the nearest facility using car, public transport and walking travel times extracted from Google Maps Platform Directions Advanced Programming Interface (Google API). The Google API is a routing service that incorporates traffic conditions and patterns (both real time and historic) and road network data to predict travel times based on a particular mode of transport between a starting and a destination location at a specified day of the week and time of the day. The starting location was populated grids of approximately 2.3 km^2^. The authors focused on driving mode (vehicular transport excluding public transport) estimates as it was the most common (70% to 78%) means to access a health facility in an emergency. They estimated the shortest travel times in high-income countries (HICs), and the longest in sub-Saharan Africa.

Second, they estimated revealed accessibility between usual residence and the nearest health facility based on anonymized smartphone usage data^16^. The resulting median travel time of 44 min was highly heterogeneous between countries. High income countries such as Singapore had the shortest travel time while low-and middle-income countries (LMICs) such as Zambia had the longest travel time. Further, the authors explored within-country inequalities based on percentile ratio of travel time. Overall countries with shorter travel times had lower within-country inequalities relative to countries with longer travel times. The authors also correlated the inequality ratio and revealed accessibility with infant mortality and life expectancy (health outcomes) at the country level. As would be expected, the accessibility metrics had a strong correlation with the health outcomes.

Third, a comparison of driving travel times between estimates of potential and realised accessibility showed a significant correlation. However, in most countries for the longest trips, there was a significant underestimation of potential travel time especially in LMICs. Finally, to understand the changes in revealed accessibility during the COVID-19 pandemic, pre-pandemic (2019) and during the pandemic (2020 and 2021) revealed accessibility metrics were compared. During the pandemic, trips to health facilities by car increased while they reduced for public transport and walking. Further, in most countries, the median travel times by car decreased, and increased for public transport and walking.

Gligorić and colleagues should be lauded for their comprehensive study. The amount of data generated is substantial. However, the authors focused on the global outlook and gave less attention to the country-level findings that would have allowed an appreciation of ground truthing (contextualize the findings relative to local knowledge within country) and policy usefulness on a country-by-country basis. Therefore, country-level policymakers may not realise the full potential of the generated outputs. While the results are useful for comparisons between countries and world regions, it masks variation within-country that would support granular policy actions^17^. Similar recent analyses were conducted at spatial resolution ranging between 20 m and 1 km^11,13,14,18^ resulting in actionable identification of hotspots (inequitable areas with relatively poorer accessibility to care)^11^. For example, in one study, specific neighborhoods or villages where travel to the nearest service provider is farther than the recommended threshold can be identified and prioritized through the use of mobile health clinics^11^.

Metrics of potential accessibility to healthcare (Box 1)^19,20^ rely heavily on observational data and often ignore the role of traffic congestion, weather variation and differences by mode of transport^20^. Such travel time estimates are rarely validated, but when they have been, they are shown as an underestimate^12^. It is only recently that potential accessibility approaches incorporating historic traffic data are being used, for example, through Google API^20^. To date, such studies have been limited to a few urban areas^20^. Gligorić et al. thus advance knowledge by deriving estimates of potential accessibility accounting for on-ground realities, globally. Revealed spatial accessibility is rarely evaluated in practice due to a lack of data on mobility^20^. Therefore, the revealed accessibility estimates provided by the authors are important in understanding actual patterns of accessibility and why they deviate from estimates of potential accessibility, statistical associations with key health outcomes, and how access to health services was modified by the pandemic.

## Box 1 A summary of approaches to measure potential accessibility to healthcare^6–9,12,15,19^

**Provider-to-population ratio**

For a given geographical area, the ratio between the provider (e.g., the number of facilities, healthworkers or beds) and the populations in need.

**Euclidean distance**

The straight-line distance between the residential area (or where the need for care is triggered) and the location of the health service provider.

**Cost distance algorithm**

Computes the cost (in terms of time or distance) of moving from a residential area to the health facility location accounting for factors that influence travel such as topography, road network, travel barriers and speeds.

**Gravity models**

Combines availability and accessibility in a defined geographic area while accounting for facility capacity and competition between facilities.

**Network analysis**

Estimates travel time or distance to a service provider along a road (transport) route.

**Self-report**

Asking either the patient or health workers to estimate the time they took to get to the service provider.

Specific limitations should be considered when interpreting the results and applying the methods, some of which the authors identified. The number of health facilities used in the analysis was incomplete. Existing global, regional, and national facility lists such as the pan-African database of health facilities^21^ and healthsites.io were not incorporated in the analysis. The WHO recently launched the Geolocated Health Facilities Data initiative to develop accurate lists per country, which we argue should also incorporate facility functionality. In addition, future spatial accessibility analyses should consider that some health services are provided in the community. For example, community vaccination campaigns, postnatal home visits, and door-to-door testing services provided by community health workers, health visitors, or accredited social health activists. Further, some healthcare services can be provided using telemedicine, and use of this modality increased exponentially during the pandemic, including for time-sensitive care such as mental health emergencies, and abortion care. Having access to an electronic device such as a smartphone or a computer, a reliable network connection, and digital literacy should be considered in future analyses which holistically consider the question of accessibility of healthcare. It is also worth noting that a facility being available does not mean that it is affordable or perceived to be of sufficient quality that patients would want to receive care there, even in an emergency. Many people in LMICs pay out-of-pocket and emergency care is sometimes a pay before service.

Despite smartphone usage by the population only recently becoming a source of mobility data, its validity has been questioned^22^. Smartphone ownership is particularly low in LMICs compared to HICs. For example, in Belgium, 90% of people had access to a smartphone (in 2021) compared to 26% in Zimbabwe (in 2020). Beyond the HIC-LMIC divide, smartphone owners are likely to be wealthier, male, educated and living in urban areas, relative to those with basic phones or no phones at all^22^. Further, only those who have resources can purchase internet connectivity and there are inherent biases to whether location history on a smartphone is enabled. In addition, mobility patterns of smartphone users differ from those without smartphones or a phone^22^. All these issues raise a question of representativeness of smartphone mobility data^22^, which may lead to biased (most likely underestimated) findings about revealed accessibility.

It is clear that estimates from routing APIs^23^ are useful in computing potential accessibility^20^ and have already been shown to reflect realistic travel times in certain settings relative to commonly used approaches^18^. However, it should be noted that the APIs are limited in rural areas where data coverage (the road network) is poor and there are few smartphone users and  motorized vehicles, meaning traffic data may be not representative. Also, many routing APIs necessitate payment for access and processing of large-scale data, and usually require prior programming expertise and access to high-speed computing infrastructure. Nevertheless, providers of APIs might consider making their tools more widely available to the research community to facilitate their use in research.

The innovative presentation of revealed accessibility by Gligorić et al. is welcome. The authors share outputs from their analysis (aggregated and anonymized). However, optimal reuse would require the proprietary raw data (location history data from mobile phone users) to be made openly accessible to other researchers, globally, who may use different analysis methods or wish to answer different research questions. Further, when using location data from mobile phones that requires a high level of granularity, ethical challenges may arise. For example, how can privacy and confidentiality of all individuals be protected while providing high resolution estimates? Both the lack of data availability and the ethical challenges might explain the small number of studies looking at revealed accessibility at regional or global scales. Alternative data sources such as patient lists^17^ and targeted surveys are more easily available and might be more useful in understanding utilization of healthcare. They will, however, require extra resources and so compete with other needs that require funding.

The authors computed travel time to the nearest facility, yet it is well established that service users commonly bypass the nearest facility even in an emergency^17^. In addition, the authors did not consider referral patterns, which tend to be relevant for health emergencies. Getting closer-to-reality travel time is just a first step toward a more comprehensive understanding of a logical path to actual care. Further, alternative transport means such as ambulances, tricycles, minivans, and motorbike taxis play an important role in emergency transport. Similarly, we need a more in-depth consideration of the concept of public transport. A publicly owned and operated integrated scheduled public transport system (buses, trams, trains, rental bikes, and cars) in a HIC is very different from a mass transport system in LMICs which is based predominantly on individual operators of vans, tuk-tuks (a simple vehicle with an engine and three wheels, often used as a taxi in some parts of the world), and minibuses—without set schedules and predictable fares and without public subsidies to continue operating during disruptions such as COVID-19. Context-specific characteristics of access need to be reflected in country-level and global estimates, particularly in LMICs which have the poorest geographic accessibility to care.

As the field moves forward, periodic assessment of healthcare accessibility using accessible, comparable datasets and innovative methods remains the cornerstone of making sure populations are not marginalised from the healthcare and life-saving interventions that they need. The innovative approach of Gligorić and colleagues using closer-to-reality travel time estimates is the future and in presenting revealed accessibility, offers many future research possibilities. Their work is not only useful as a methodological contribution to spatial accessibility research, but more importantly, a potential high-impact advocacy tool to push governments and policymakers to recognize and address poor accessibility. However, to truly get to policy-relevant spatial assessments of health care services, more collaboration amongst pertinent players, open access data, and granularity of evidence presentation will be critical.
