# Author Correction: Advancing the frontiers of geographic accessibility to healthcare services

**DOI:** 10.1038/s43856-024-00501-2

**Published:** 2024-04-23

**Authors:** Peter M. Macharia, Aduragbemi Banke-Thomas, Lenka Beňová

**Affiliations:** 1 Department of Public Health, Institute of Tropical Medicine, Antwerp, Belgium; 2 Population & Health Impact Surveillance Group, Kenya Medical Research Institute-Wellcome Trust Research programme, Nairobi, Kenya; 3 Faculty of Epidemiology and Population Health, London School of Hygiene and Tropical Medicine, London, UK; 4 Maternal and Reproductive Health Research Collective, Lagos, Nigeria; 5 School of Human Sciences, University of Greenwich, London, UK

**Keywords:** Epidemiology, Epidemiology

Correction to: *Communications Medicine* 10.1038/s43856-023-00391-w, published online 03 November 2023

In the original version of this article, the DOI for reference 16 was incorrect. The reference was ‘Gligorić K. et al. Revealed versus potential spatial accessibility of healthcare and changing patterns during the COVID-19 pandemic. *Commun. Med*. 10.1038/ncomms10535 (2023)’. It has now been corrected to ‘Gligorić K. et al. Revealed versus potential spatial accessibility of healthcare and changing patterns during the COVID-19 pandemic. *Commun. Med*. 10.1038/s43856-023-00384-9 (2023)’. This correction has been made in the HTML and PDF versions of this article.

